# Sea Voyages

**Published:** 1878-02

**Authors:** 


SEA VOYAGES, and the best means of enjoying them; and first of all have a plenty of woolen clothing and wear it even in midsummer except during the middle of still days ; but every day, and all day a good flannel shirt should be worn next the skin even in the tropics, to counteract the baleful effect of damps, fogs, and changes of temperature. The British government compels its sailors to wear woolen flannel shirts all the year round in all latitudes as a result of its observed necessity in keeping off disabling diseases.

